# Magnitude of diabetes self-care practice and associated factors among type two adult diabetic patients following at public Hospitals in central zone, Tigray Region, Ethiopia, 2017

**DOI:** 10.1186/s13104-018-3489-0

**Published:** 2018-06-13

**Authors:** Teklewoini Mariye, Hagos Tasew, Girmay Teklay, Hadgu Gerensea, Workinesh Daba

**Affiliations:** 1 Department of Adult Health Nursing, School of Nursing, College of Health Science and Referral Hospital, Aksum University, Tigray, Ethiopia; 2 Department of Pediatric Nursing, School of Nursing, College of Health Science and Referral Hospital, Aksum University, Tigray, Ethiopia; 3 Department of Nursing and Midwifery, School of Allied Health Science, College of Health Science, Addis Ababa University, Addis Ababa, Ethiopia

**Keywords:** Self-care, Practice, Factors, Diabetes mellitus, Ethiopia

## Abstract

**Objective:**

Self-care practice in type two diabetes is a critical factor to keep the disease under control. Despite the important role of self-care practices in management of diabetes were recognized to be useful and effective in achieving diabetes control and preventing its complication, findings of previous studies in Ethiopia were confirmed that, aspects of self-care practices were more problematic. So that this study focus on magnitude of self-care practice and associated factors among diabetic patients.

**Results:**

Among the total 284 respondents, their mean age was 52.19 years and about 178 (62.7%) had poor diabetic self-care practice. Having glucometer at home (AOR = 3. 719 [1.700, 8.139]), knowing fasting glucose level (AOR = 2. 709 [1.481, 4.957]), attending diabetic education (AOR = 2. 487 [2.027, 6.020]), perceived benefit (AOR = 2. 422 [1.374, 4.269]), perceived barrier (AOR = 0. 471 [0.265, 0.394]), and self-employment (AOR = 5. 936 [1.965, 17.936]) were significantly associated with good self-care practice.

## Introduction

Self-care practice in diabetes patient is a critical factor to keep the disease under control and about 95% of the disease management is usually carried out by the affected individual or their families [1].

Diabetes mellitus and its complications bring extensive economic loss in diabetic patients with their family, to health systems and to the national economies through direct medical costs and loss of work and incomes [2].

In developing countries, where the resources are limited, and treatment costs of diabetes are constantly increasing, the practice of self-care component among patients with diabetes may result in better economic and therapeutic outcomes [3].

Consistent implementation of recommended self-care behaviors in individuals with type two diabetes requires collaboration between the patient and the provider in an enabling health care system with adequate facilities and resources [4]. This is a major challenge for many sub-Saharan countries in the wake of the rising prevalence of diabetes because sub-Saharan Africa is faced with inadequate facilities/resources, inadequately skilled staff, and lack of resources for diabetes education [5, 6].

Despite the important role of self-care practices in management of diabetes were recognized to be useful and effective in achieving diabetes control and preventing its complication, findings of previous studies in Ethiopia were confirmed that, aspects of self-care practices were more problematic [7–9].

There is no study conducted in Ethiopia specifically in Tigray Region regarding self-care practice on diabetic patients and the few studies conducted in developing countries have also discrepancy on the self-care practice among diabetes patients. Therefore, this study focus on magnitude of self-care practice and associated factors among diabetic patients.

## Main text

### Study area and period

The study was conducted in public Hospitals of central zone, Tigray region, Ethiopia. The research was conducted from April 1 to March 1 2017.

### Study design

A Hospital based cross-sectional study design was conducted.

### Source populations

Were all type two diabetic patients who visits general public Hospitals of the central zone of Tigray, Ethiopia.

### Study populations

Were all type two adult DM patients who visit general public Hospitals of the central zone of Tigray at the time of the data collection period.

### Eligibility criteria

We were excluded critical ill patients from the study participants.

### Sample size determination

Total sample size was calculated using an EPI Info software version 7.1.1 with the parameters for the single proportion formula. Confidence level, 95%, marginal error (d) 5% and 56.6% prevalence of self-care practice was taken from a study conducted in TASH, Addis Ababa in 2012 [7]. Assuming a non-response rate of 10% the final sample size was 284.

### Sampling technique

A systematic random sampling technique was employed, and every three patients were selected and the first patient drawn randomly.

### Data collection instrument and techniques

Data was collected using a semi-structured interview administered questionnaire which had five subparts, namely social, demographic, clinical characteristic, knowledge related questions, health belief questions and self-care practice related questions. Knowledge and practice questions were adapted from media USA [8, 10, 11]. The diabetic health belief was assessed by adapting as developed by Given [12, 13]. The questionnaire was initially prepared in English then translated into the local language (Tigrigna) by an individual who has good ability of the two languages then translated back to English by different person to ensure consistency. A week prior to the actual data collection period, the questionnaire was pre-tested on 5% of the total sample size patients identified from Suhul Hospital which is found in the Northwest zone of Tigray, Ethiopia. Data was collected by four trained B.Sc. nurses and two supervisors (B.Sc. nurses).

### Data processing and analysis procedures

The data were checked for its completeness manually and then entered in Epi date version 3.1 and analyzed using SPSS version 22 statistical software package. A bivariate logistic regression model analysis was done to see the association between the explanatory and outcome variables. Henceforth, multivariable logistic regression analysis was employed by selecting only variables with *P* value ≤ 0.2 in the bivariate analysis. To check for goodness of fit the model, the Hosmer–Lemen show test was used. Multicollinearity was assessed by variance inflation factor and all assumptions of binary logistic regression were checked. Odds ratio with 95% CI was used to measure the strength between dependent and independent variables at P-Value < 0.05 to determine the level of statistical significance.

### Study variables

#### Dependent variable

- Diabetic self-care practice.

#### Independent variable

*Socio-demographic factors* Age, sex, religion, educational, occupational, income and marital status.

*Clinical characteristic* Duration of DM, family history of diabetes, types of treatment, comorbidity, member of diabetic association, attending diabetic education.

*Diabetes health belief* Perceived susceptibility, severity of DM, Perceived benefits and Perceived barrier.

*Diabetic knowledge* Knowledge about DM, Knowledge of diabetes self-care practices.

### Operational definitions

*Good self-care practice* Is those who scored the mean and above the overall self-care Practice score [10, 14, 15].

*Poor self-care practice* Is those who scored below the overall mean self-care practice score [10, 14, 15].

### Results

#### Clinical characteristics and knowledge

The mean duration of diabetes patients was 5.03 (95% CI) with minimum of 1 year and a maximum of 24 years. Of the total respondents, 91 (32%) of the total respondents had a family history of diabetes, and only 44 (15.5%) respondents had a glucometer at home. From all of the respondents, only 98 respondents (9.9%) attended diabetic education. Of the total respondents, only 98 of the respondents (34.5%) were member of diabetic association and 98 (34.5%) were did not know on the presence of Ethiopian diabetic association (see Table 1).

**Table 1:** Clinical characteristics and knowledge among type two DM at public Hospitals, Central Zone, Tigray, Ethiopia, 2017 (n = 284)

Variables	Category	Poor practice N = (%)	Good practice N = (%)	Total N(%)
Duration of the disease	≥ 5 years	118 (66.3%)	78 (73.6%)	196 (69%)
	> 5 years	60 (33.7%)	28 (26.4%)	88 (31%)
Comorbidity	Yes	59 (33.1%)	54 (50.9%)	113 (39.8%)
	No	119 (66.9%)	52 (49.1%)	171 (60.2%)
Family history of diabetes	Yes	45 (25.3%)	46 (43.4%)	91 (32%)
	No	133 (74.7%)	60 (56.6%)	193 (68%)
Attended diabetic education	Yes	12 (6.7%)	16 (15.1%)	28 (9.9%)
	No	166 (93.3%)	90 (84.9)	256 (90.1%)
Member of diabetic association	Yes	47 (26.4%)	51 (48.1%)	98 (34.5%)
	No	73 (41%)	37 (34.9%)	110 (38.7%)
	I did know	58 (32.6%)	18 (17%)	76 (26.8%)
Do you have your own glucometer	Yes	15 (8.4%)	29 (27.4%)	44 (15.5%)
	No	163 (91.6%)	77 (72.6%)	246 (84.5%)
Knowledge	Poor knowledge	79 (44.4%)	47 (44.3%)	126 (44.4%)
	Good knowledge	99 (55.6%)	59 (55.7%)	158 (55.6%)

#### Diabetic health belief model

Diabetic health belief was assessed using the perceived susceptibility, perceived severity, perceived benefits and barriers to self-care practice. Accordingly, more than half study participants (58.8%) reported favorable attitude to perceived susceptibility and more than half of the respondents (49.3%) had a favorable attitude to perceived benefits and barriers (see Table 2).

**Table 2:** Health belief model among type two DM at public Hospitals, central zone, Tigray, Ethiopia, 2017 (n = 284)

Variables	Category	Poor practice N (%)	Good practice N (%)	Total
Perceived susceptibility	Unfavorable attitude	65 (36.5%)	52 (49.1%)	117 (41.2%)
	Favorable attitude	113 (63.5%)	54 (50.9%)	167 (58.8%)
Perceived severity	Unfavorable attitude	86 (48.3%)	62 (58.5%)	148 (52.1%)
	Favorable attitude	92 (51.7%)	44 (41.5%)	136 (47.9%)
Perceived benefit	Unfavorable attitude	104 (58.4%	40 (37.7%)	144 (50.7%)
	Favorable attitude	74 (41.6%)	66 (62.3%)	140 (49.3%)
Perceived barrier	Unfavorable attitude	71 (39.9%)	61 (57.5%)	132 (46.5%)
	Favorable attitude	107 (60.1%)	45 (42.5%)	152 (53.5%)

#### Factors associated with diabetes self-care practice

In the multivariable logistic regression analysis, only sex variables had shown an overall significant effect on good self-care practice at the 5% level of significance.

Having glucometer at home showed significant association with good self-care practice. The odds of having glucometer at home were 3.7 times more associated with good self-care practice than to those who were not having glucometer at home (AOR = 3. 719; 95% CI [1.700, 8.139]). Knowing glucose level was significantly associated with good self-care practice. This study showed that those who were knew glucose level were 2.7 times more likely associated with good self-care practice than to those who did not know their glucose level (AOR = 2. 709; 95% CI [1.481, 4.957]).

Attending diabetic education was significantly associated with good self-care practice. Patients who were attending diabetic education were 2.5 times more associated with good self-care than to their counterpart (AOR = 2. 487; 95% CI [2.027, 6.020]).

Those who had positive attitudes to perceived barriers had significant association with the outcome variable of good self-care practice. Those favorable attitudes to perceived barriers were less associated with good self-care practice than to their counterpart by 52.9% (AOR = 0. 471 CI [0.265, 0.394]).

Those who had favorable attitudes to perceived benefits had significant association with the outcome variable of good self-care practice. Those favorable attitudes to perceived benefit were 2.4 times more associated than those unfavorable attitudes to good self-care practice (AOR = 2. 422 CI [1.374, 4.269]).

Those who, recruited as self-employed had significant association without income variable good self-care practice. Those who, recruited as self-employed were 5.936 times more associated with good self-care practice than those unemployed to good self-care practice (AOR = 5. 936, 95% CI [1.965, 17.936]) see Table 3).

**Table 3:** Logistic regression among type two DM at public Hospitals, central zone, Tigray, Ethiopia, 2017 (n = 284)

Variables	Category	Poor practice	Good practice	COR [95% CI]	AOR [95% CI]
Occupation	Unemployed	50 (28.1%)	28 (26.4%)	1	1
	Employed	30 (16.9%)	16 (15.1%)	0.952 (0.444, 2.043)	0.764 (0.310, 1.885)
	Self employed	15 (8.4%)	21 (19.8%)	2.500 (1.114, 5.609)	5.93 (1.965, 17.936)*
	Housewife	6 (3.4%)	3 (2.8%)	0.893 (0.207, 3.849)	1.481 (0.268,8.183)
	Farmer	77 (43.3%)	38 (35.8%)	0.881 (0.482, 1.612)	2.014 (0.862, 4.705)
Attend education	Yes	12 (6.7%)	16 (15.1%)	2.459 (1.115, 5.425)	2.48 (1.027, 6.020)*
	No	166 (93.3%)	90 (84.9)	1	1
Member association	Yes	47 (26.4%)	51 (48.1%)	3.496 (1.806, 6.771)	1.641 (0.764, 3.526)
	No	73 (41%)	37 (34.9%)	1.633 (0.844, 3.161)	
	I did know	58 (32.6%)	18 (17%)	1	1
Glucometer	Yes	15 (8.4%)	29 (27.4%)	4.093 (2.074, 8.076)	3.719 (1.700, 8.139*
	No	163 (91.6%)	77 (72.6%)	1	1
Current FBS	Yes	65 (36.5%)	65 (61.3%)	2.756 (1.679, 4.525)	2.707 (1.48, 4.957)*
	No	113 (63.5%)	41 (38.7%)	1	1
	Favorable attitude	74 (41.6%)	66 (62.3%)	2.319 (1.416, 3.797)	2.42 (1.374, 4.269)*
Perceived barrier	Unfavorable attitude	71 (39.9%)	61 (57.5%)	1	1
	Favorable attitude	107 (60.1%)	45 (42.5%)	0.490 (0.300, 0.798)	0.471 (0.265, 0.838)*

*Significant association at P-value less than 0.05

### Discussion

The magnitude of overall good diabetes self-care practice was 106 (37. 3%) among type two diabetic patients on follow-up at a public Hospitals in the central zone of Tigray. This study was consistent with the study conducted in Harari Town in (2012) (39%) [9] and Felege Hiwot referral Hospital in (2013) (36%) [8] and study conducted in Kenya in 2010 (41%) [16]. But this study is lower than with a study conducted in Addis Ababa Public Hospitals in (2016) [17] Dilla University Hospital in (2014) [18] and Nekemte referral Hospitals in 2013 [18] which were 60.3, 56, 76.8 and 55% respectively. This variation might be due to, socio cultural variation, life style difference, inadequate access to glucose monitoring machines, test strips and level of education of the general public.

Having glucometer at home showed significant association with good self-care practice. The odds of having glucometer at home were 3.7 times more associated with good self-care practice than compared to those who were not having glucometer at home. This study was similar to a study conducted in TASH in (2012) [7] which indicated having glucometer at home was two times more associated with good self-care practice. These could be having glucometer at home might reinforce patients to measure and to control their blood glucose level regularly, which leads to change life style that promote good self-care practice.

Knowing glucose level was significantly associated with good self-care practice. This study showed that those who knew fasting glucose level were 2.7 times more associated with good self-care practice than their counterpart. This might be due to a patient encourages to perform essential self-care behaviors such as healthy eating, being physically active, risk reduction behavior and also it encourages to apply the recommended action to self-care practice.

Attending diabetic education was significantly associated with good self-care practice. Patients who were attending diabetic education were 2.5 times more likely associated with the outcome variable than to their counterpart. This study is consistent with studies conducted in Addis Ababa Public Hospitals in (2016) [17] in those who usually receive education, from health care professionals were almost three times more associated with good self-care practice than those who did not usually receive education from health professionals. This might be education stimulates the individual’s performance of diabetes self-care to increase target behavior such as blood glucose monitoring, diet care, physical activity and medical care among adults.

Those who had favorable attitudes to perceived benefit had significant association with the outcome variable of good self-care practice. Those favorable attitudes to perceived benefits were 2.4 times more associated with good self-care practice than to their counterpart. This study was consistent with a study conducted in Nigeria in (2014) [13] 12.4 times more associated with good self-care practice. This might be due to having Positive beliefs about expected benefits are usually shown as an essential factor in doing a special health behavior self-care practice activity.

Those who had favorable attitudes to the perceived barrier had significant association with the outcome variable of good self-care practice. Those favorable attitudes to the perceived barrier were less likely associated with good self-care practice than to the opposite side by 52.9%. This study is consistent with studies conducted in Harare Town in (2012) [9] less likely associated with the outcome variable by 70%. This might be due to poor adherence to self-care activity due to unfavorable attitude toward the disease and level of education the general public.

Those who, recruited as self-employed were 5.9 times associated with good self-care practice than those unemployed to good self-care practice. This study is consistent with study conducted in Nekemte referral Hospital in 2014 [19] around four times more associated. This similarity might be due to non-financial barrier, have favorable attitude to self-care practice activity and Self-employment can associated both with good life evaluation and good emotional well-being.

### Conclusion

This study established that, more than half of respondents had poor self-care practice. So that, Health care personnel and Ethiopian diabetic association must increase the patient’s awareness to the importance of self-care practices domains and strongly promote the practice among diabetes patients by strengthening IEC program.

## Limitations

- There may have been recalled bias and social desirability bias since the self-care practices of the study participants were based on self-reports and performance of these behaviors were not observed and could not be confirmed.
- The study design was cross-sectional, the direction of causal relationships between variables can’t always determine.

## Abbreviations

ADA: American Diabetic Association
AOR: adjusted odd ratio
COR: crudes odd ratio
DM: diabetic militias
IEC: information education and communication
SPSS: Statistics Package for Social Science
DM2: type two diabetic militias
WHO: World Health Organization
TASH: Tikur Anbesa Specialized Hospital
TRHB: Tigray Regional Health Burro
